# Traumatic hemifacial avulsion and degloving injury: a case report

**DOI:** 10.11604/pamj.2022.41.158.32802

**Published:** 2022-02-23

**Authors:** Alphonce Nsabi Simbila, Paulo Joseph Laizer, William Njoroge Waihenya, David Kiwango Deoglas, Audrey Bernard Mwashilemo, Dorah Jonathan Kiwale

**Affiliations:** 1 Department of Emergency Medicine, Muhimbili National Hospital, Dar es Salaam, Tanzania,; 2 Department of Oral and Maxillofacial Surgery, Muhimbili University of Health and Allied Sciences, Dar es Salaam, Tanzania,; 3 Department of Emergency Medicine, Muhimbili University of Health and Allied Sciences, Dar es Salaam, Tanzania,; 4 Department of Ophthalmology, Muhimbili University of Health and Allied Sciences, Dar es Salaam, Tanzania,; 5 Department of Oral and Maxillofacial Surgery, Muhimbili National Hospital, Dar es Salaam, Tanzania

**Keywords:** Face, degloving, avulsion, maxillofacial injury, case report

## Abstract

Gross maxillofacial injuries are challenging to manage because they can be complicated by airway obstruction, injuries to the cervical spine, and cranial structures. Deformities from such injuries have lasting psychological effects which if not addressed can be devastating. We present a 21-year-old male motorcyclist who was involved in a motor traffic collision and sustained avulsion and degloving of the forehead skin, left eyebrow, left upper and lower eyelids, the nose, the left cheek and part of the right cheek, upper and lower lips, and the skin overlying the chin. His airway was compromised; hence rapid sequence intubation was done to secure it. Thereafter single-stage primary reconstruction and repair were done. A multidisciplinary team approach involving different specialties yielded good outcomes for this patient's condition.

## Introduction

Severe facial injuries can result from high-speed motor vehicle collisions, assault and domestic violence, animal bites, and falls. They pose significant management challenges due to their associated morbidity and potential mortality. Extensive facial avulsion and degloving injuries resulting from such mechanisms of injury carry huge possibilities of airway compromise, profuse bleeding, soft tissue loss, severe disfigurement, and subsequent post-traumatic aesthetic changes and functional limitations. Deformities resulting from such injuries have long-lasting psychological effects which if not addressed can be devastating [1-3]. However, in the early hours of the encounter with such injuries clinicians should center their attention on the basics of trauma care. Once life-threatening conditions have been taken care of an emergency physician has ample time to either close facial wounds or consult other specialties. Location of the wound, size, depth, shape, time for successful aesthetic wound closure, and resource availability are among the factors an emergency physician should consider when making decisions to close the wound. We present a case of a 21-year-old male motorcyclist with extensive traumatic facial avulsion and degloving injuries involving half of his face following a motor traffic crash. We emphasize the importance of early maintenance of the airway, bleeding control, stabilization, damage control, and timely multispecialty involvement in surgical reconstruction for better outcomes.

## Patient and observation

**Patient information:** a 21-year-old male motorcyclist was referred to the emergency department of Muhimbili National Hospital with facial injuries 6 hours after being involved in a collision with a fast-moving car. He had actively bleeding avulsion and degloving facial injuries involving mostly the left half of the face and alteration in mentation.

**Figure 1:**
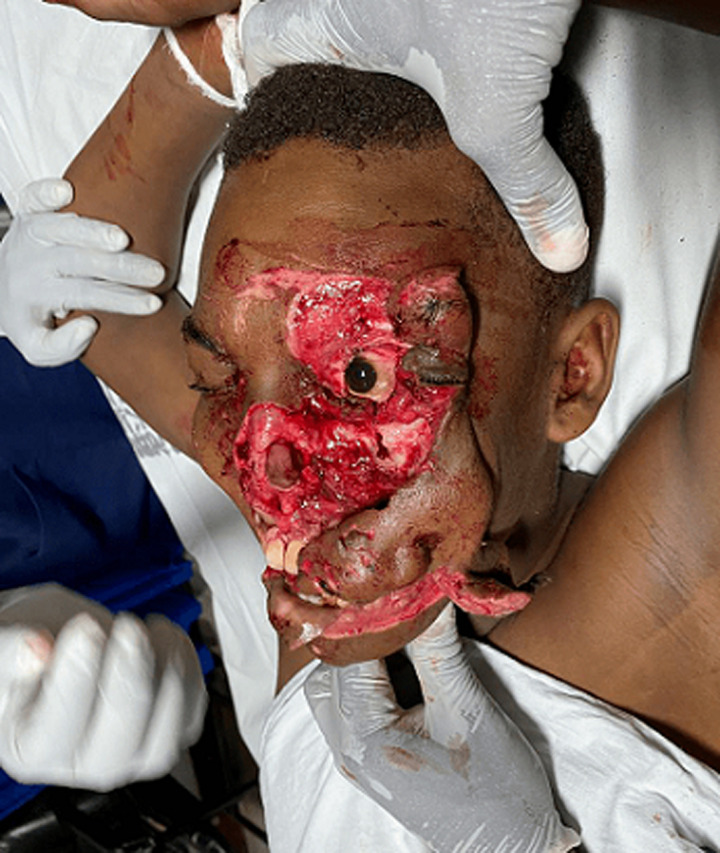
initial avulsion and degloving injury of the face before rapid sequence intubation

**Clinical findings:** on initial assessment at the emergency department, the patient had a blood pressure of 91/41mmHg, pulse rate of 130 beats/min, respiratory rate of 28 breaths/min, oxygen saturation of 99% on room air, random blood glucose of 11.6mmol/L, and his body temperature was 35.8°C. His glasgow coma score was 13/15. The airways were compromised by blood from the facial injuries and detached flaps of soft tissue blocking them. There was avulsion and degloving of the left side of the face including the forehead skin, left eyebrow, left upper and lower eyelids, the nose, the whole of the left cheek, and part of the right cheek, upper and lower lips, and the skin overlying the chin (Figure 1). The left eye globe, sight, and extraocular movements were intact.

**Timeline of the current episode:** 21^st^ November, 2021: the patient presented to the Emergency Department of Muhimbili National hospital with head and facial injuries. Endotracheal intubation and other diagnostic investigations were done. Within 45 minutes, he was taken to the emergency operating theater for primary reconstructive surgery of his facial avulsion and degloving injuries together with the eyelids repair. He was sent to the surgical intensive care unit after the surgery. 22^nd^ November, 2021: the patient was successfully weaned off the ventilator, extubated, and continued with post-operative care in the surgical intensive care unit with no untoward events. 24^th^ November, 2021: the patient was stable and was transferred to the oral and maxillofacial ward for further care. 30^th^ November, 2021: surgical sutures were removed. 5^th^ December, 2021: patient was discharged from the hospital and requested to attend an oral and maxillofacial clinic for follow up.

**Diagnostic assessment:** point of care Extended Focused Assessment with Sonography in Trauma (E-FAST) was negative. A computed tomography (CT) scan of the head and cervical spine showed a scalp hematoma, a non-displaced left anterior maxillary fracture with haemosinus, and a small epidural hematoma on the left frontal lobe. The cervical spine was normal. X-rays of the pelvis left distal tibia-fibula and ankle were normal. His pre-operative hemoglobin level was 10.4 g/dL and platelets were 231.3K/µL.

**Diagnosis:** polytrauma with facial avulsion and degloving wounds, open maxillary fracture, mild traumatic brain injury and cervical spine injury, in impending airway obstruction and hemorrhagic shock.

**Figure 2:**
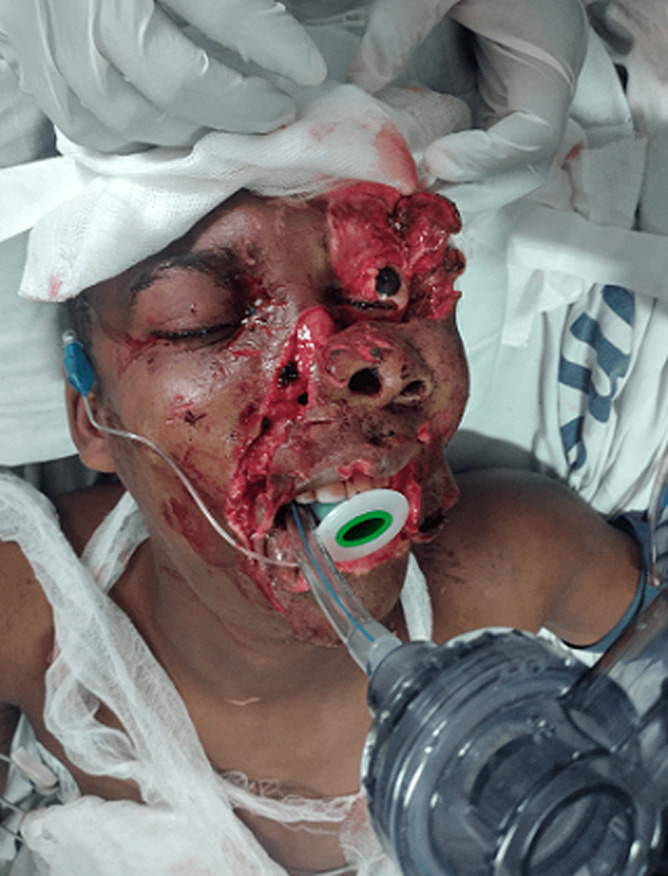
examination of injuries after the establishment of the definitive airway by rapid sequence intubation with an endotracheal tube and initial stabilization

**Figure 3:**
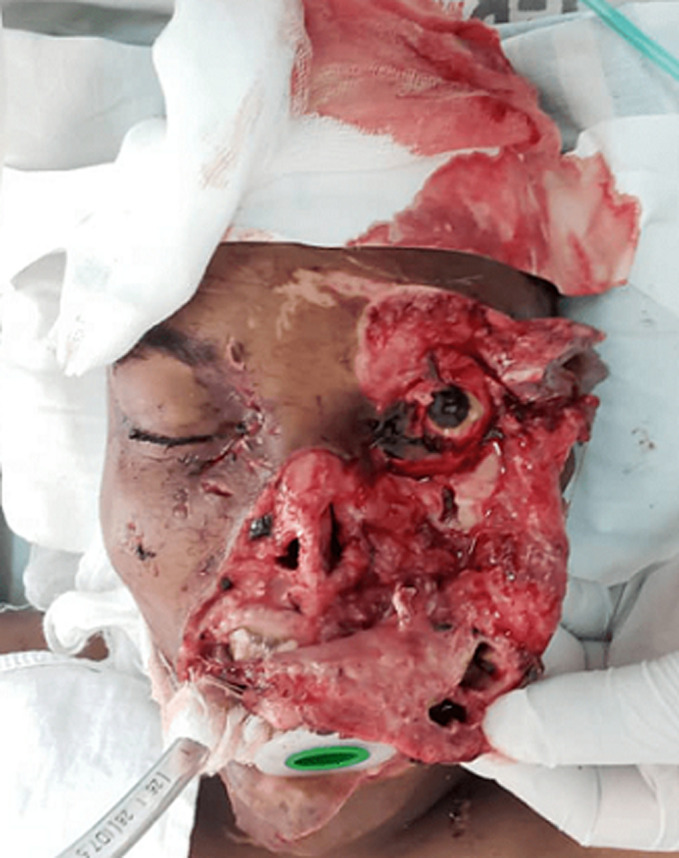
overall examination of the avulsion and degloving injuries under anesthesia after cleaning and debridement was done in theatre

**Figure 4:**
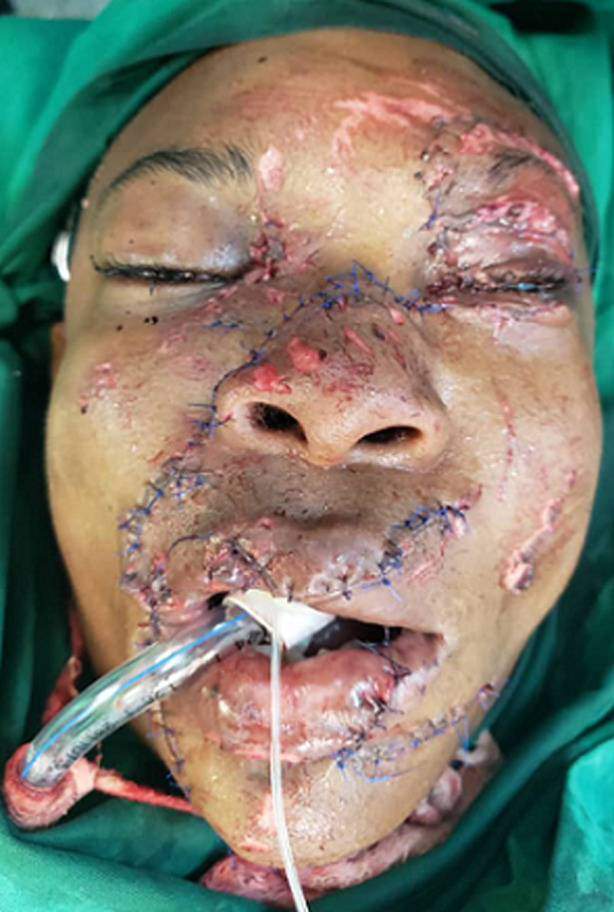
intra-operative condition of the patient's face soon after the replacement and repair of the injured structures

**Therapeutic interventions:** rapid sequence intubation to protect the airway was done at the emergency department (Figure 2). Two liters of intravenous normal saline and 2 units of whole blood were given to optimize circulation. Pre-operatively, intravenous Ceftriaxone (1g stat) and Metronidazole (500mg stat) were initiated. He was then taken into the operating theatre. Under general anesthesia, the skin was prepared with povidone-iodine and wounds irrigated with normal saline and diluted betadine iodine (Figure 3). Meticulous suturing was done to restore each of the facial structures to their proper anatomical position. Deep tissues of the oral mucosa, upper and lower lip, chin and commissure, lateral nose, and nasal base were repaired using absorbable suture whereas for the skin nylon suture was used. The nose was repaired in layers starting with the mucosal lining and cartilage framework. The right nasal ala was then repositioned and the skin sutured in place (Figure 4). The right medial eye canthal laceration and avulsion were sutured in place in layers. In the left eye, inner structures such as the lacrimal and tarsal glands were arranged and sutured in place in layers as well. The eyelid laceration margins were apposed and sutured. Then the rest of the skin was repositioned and sutured. Hemostasis was achieved. The eye was padded with tetracycline eye ointment.

**Figure 5:**
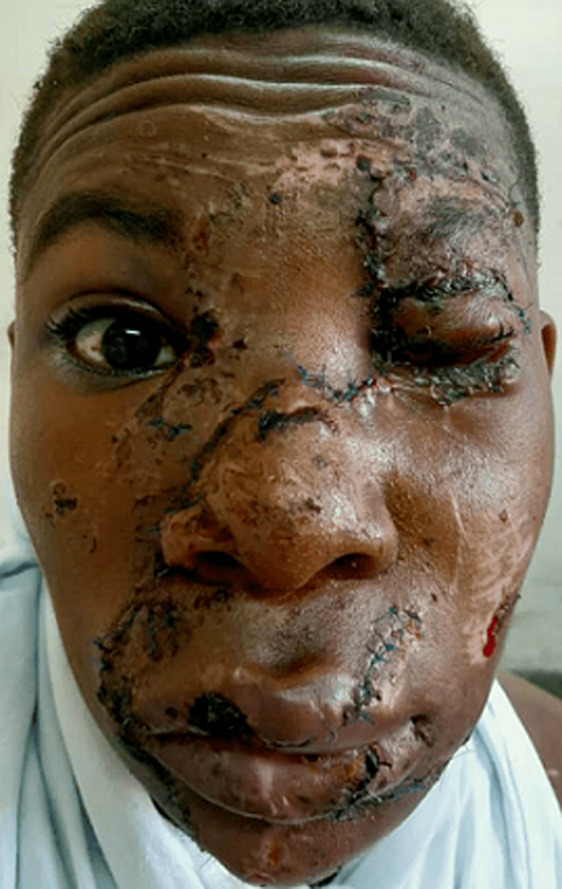
healing wounds with sutures in position after the single-stage primary repair

**Figure 6:**
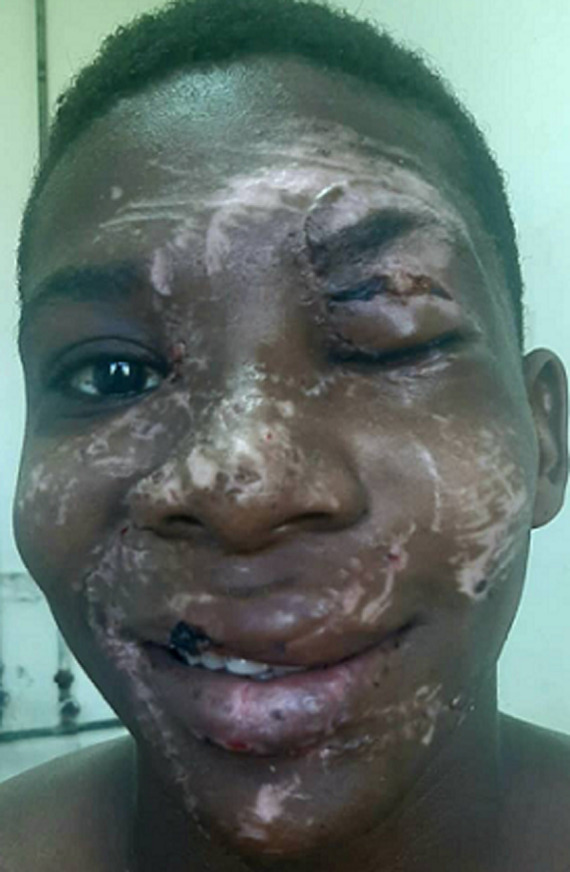
well repaired and positioned facial structures in the progressive healing process after suture removal

**Follow-up and outcome of interventions:** post-operatively, the patient spent 3 uneventful days in the surgical intensive care unit before being shifted to the oral and maxillofacial ward. While in the ward he continued receiving pain medications, antibiotics, fluids, and wound care. Sutured facial wounds were progressively healing well without any tissue necrosis (Figure 5, Figure 6). An ophthalmology review was done and determined that he had no problems with visual acuity.

**Patient perspective:** I don´t remember any details of what happened. My mother kept telling me how bad the injuries were at the time they brought me to the hospital. Everybody thought I would not make it. Then, a week after, when I was in the ward and was able to walk and talk properly, the emergency physician who received me on the day of the injury came to talk to me, I asked him to show me the pictures of the injuries he took before they were repaired. When I first saw them, I felt like crying but was full of gratitude. I don´t know how I survived such injuries. The doctors narrated how they treated me initially by putting a tube in my throat to support air entry into my lungs and the facial surgery later in the operating theatre. I then saw my pictures after they had been repaired, I was filled with awe. I thank all the doctors who saved my life. They re-created my face and gave me a second chance to live. I thank God, I now know He loves me. Victims of accidents like me should be able to get this kind of service everywhere in our country. Let my experience and all the services I got to save me be used to teach others.

**Informed consent:** written informed consent was obtained from the patient for publication of this case report, accompanying images, and de-identified information. A copy of the written consent is available for review.

## Discussion

Gross maxillofacial injuries are extremely challenging to manage because they can be complicated by the presence of airway obstruction and concomitant injuries to the cervical and cranial structures. However, physicians must focus on the principles of trauma care to promptly recognize life-threatening conditions and intervene appropriately. Patients with serious avulsion and degloving facial injuries may not be able to protect their airways due to their deformed anatomy and bleeding. Soft tissue flaps can act as a foreign body in their airway. These patients usually end up being intubated early in the course of their condition [4].

In serious facial injuries, the primary concern of an emergency physician is the maintenance of the airway. Facial injuries make ventilation with bag-valve-mask almost impossible. The presence of blood on the face together with the deformities such as fractures of the mandible and the midface breach the normal seal of the mask to the face during non-invasive ventilation. Associated neck injuries with the tracheal distortion can make it difficult to locate the anatomical landmarks for cricothyrotomy. Even in the hands of the most skilled physician, establishment and maintenance of a definitive airway either by an endotracheal tube or surgical airway can be challenging [5]. Deformities and ongoing bleeding encountered in our patient made ventilation by bag-valve-mask impossible. A cricothyrotomy tray and a video laryngoscope were prepared in anticipation of a difficult airway. After a few rounds of suctioning and repositioning of the avulsed soft tissue, we were able to intubate the patient successfully.

Repairing extensive and overlapping facial avulsion and degloving wounds requires a multidisciplinary approach. Plastic surgeons, otorhinolaryngologists, oral and maxillofacial surgeons, and ophthalmologists may be involved early in the management plan. In the management of our patient, we made early consultations with the ophthalmologist, oral and maxillofacial surgeons when the initial assessment revealed lacerations which included eyelid margins, cartilage damage, large flaps, nasal wounds, and other deep facial structures. This team approach ensured timely subspecialty planning of primary repair, decision making and thus achieved favorable reconstructive results for the patient [6]. Early single-stage primary reconstruction provides good functional and cosmetic outcomes [7,8]. Wounds with contamination or embedded material are cleaned, debrided, and repaired without wasting time. Caution is taken to try and preserve as much original tissue as possible while doing the debridement. Doing so prevents infection and flap loss. Better results such as less infection and scarring are achieved with immediate soft tissue reconstruction. Our patient´s reconstruction was performed within 6 hours of injury which increased the chances of better outcomes.

Repair of eyelid lacerations needs ophthalmologic attention since they may involve structures such as the lacrimal gland and canalicular drainage system. Their repair can be done within 48 hours of injury. Our patient had lacerations involving the medial canthus, the lacrimal, and the tarsal glands which got ophthalmology review and repair within the required time. Primary detailed closure of eye-lid margins in layers ensured good results [9]. Even though debridement and proper washing of the wounds were done our patient required prophylactic antibiotics because his wounds were grossly contaminated, exposed the nasal cartilage, and were associated with a non-displaced open maxillary fracture. He received a broad-spectrum antibiotic coverage for gram-negative, gram-positive, and anaerobic bacteria. He was also given a shot of tetanus toxoid because his tetanus immunization schedule was unknown [10]. Non-displaced and minimally displaced maxillary fractures usually do not need emergent definitive care. Otherwise, the definitive repair is done within a week. Our patient had a non-displaced left maxillary fracture which did not pose any functional or cosmetic concerns. It was conservatively and successfully treated with analgesia.

## Conclusion

Extensive facial avulsion and degloving injuries due to trauma do not occur in isolation. They can potentially be complicated by airway obstruction, cervical and brain injuries. Sometimes their management requires a set of skills beyond basic trauma care, hence the involvement of other specialties should be considered early. Soon after life-threatening conditions have been taken care of early single-stage primary reconstruction and repair provide good functional and cosmetic outcomes.
